# Life after lockdown: The experiences of older adults in a contactless digital world

**DOI:** 10.3389/fpsyg.2022.1100521

**Published:** 2023-01-13

**Authors:** Benjamin A. Morrison, James Nicholson, Becca Wood, Pam Briggs

**Affiliations:** ^1^Psychology and Communication Technology Lab, Department of Psychology, Northumbria University, Newcastle upon Tyne, United Kingdom; ^2^School of Criminology, Simon Fraser University, Burnaby, BC, Canada

**Keywords:** aging, older adults, technology, COVID-19, digital exclusion, digital divide

## Abstract

**Introduction:**

The digital response to the Coronavirus (COVID-19) pandemic and its effects on the lives of older adults has been well-documented, but less is known about how they experienced the post-lockdown re-emergence into a relatively contactless digital society.

**Methods:**

We report the findings from a qualitative survey (*n* = 93) and subsequent interviews (*n* = 9) with older adults aged 50+, where they describe their struggles with some of the newly implemented digital interactions. These struggles cover a range of settings but include using contactless payments, QR codes and apps to facilitate transactions in cafes, bars, and restaurants.

**Results:**

A thematic analysis of our data revealed the intrinsic (e.g. digital literacy) and extrinsic (e.g. malfunctioning technology) factors that limited social inclusion for these participants, and that sometimes even led to moments of public humiliation.

**Discussion:**

Our findings shed light on some of the motivational factors that underpin the age-related digital divide, whilst also highlighting the role of self-directed agism in limiting motivations to learn new digital routines.

## 1. Introduction

The Coronavirus (COVID-19) crisis was accompanied by a rapid digital revolution. Within the home, lockdown periods triggered a move to working from home and homeschooling, placing a new digital burden on families (Shek, 2021), while social activities with friends and family moved online (Hantrais et al., 2021; Shek, 2021). Outside of the home, contactless technology became ubiquitous, with a major growth in cashless and touchless interactions *via* smartcards, smartphones, QR codes, or other forms of seamless digital exchange (Huterska et al., 2021; Iqbal and Campbell, 2021). In many countries, there was a mass rollout of apps and digital certificates designed to control the spread of COVID-19 by restricting access to those who were not vaccinated or issuing digital notifications for individuals who had come into close physical contact with those who were infected. In short, there was a mass “digital first” response to many of the problems created by the pandemic, but this also meant that many existing digital inequalities were exacerbated, with older adults, ethnic minorities, the disabled, and those of lower socioeconomic means particularly disadvantaged (Hantrais and Letablier, 2020; Litchfield et al., 2021; Nguyen et al., 2021; Poole et al., 2021).

Older adults faced significant challenges during this period. Firstly, they were more likely to experience serious health consequences if exposed to COVID-19. Secondly, COVID-19 became seen as the “older adult problem” with older adults sometimes vilified by those objecting to social distancing measures (Lichtenstein, 2021), while the most vulnerable were asked to shelter indoors for long periods (Fraser et al., 2020). Many older adults struggled as people turned to digital means to stay in touch with friends (Haase et al., 2021), shop for food and basic necessities (Palmer et al., 2021), and access healthcare (Choi et al., 2022). Some already had the requisite digital skills and others quickly acquired them (Xie et al., 2020; Kim et al., 2022); however, many others faced what Seifert et al. (2021) called the “double burden of social and digital exclusion.” Although defining older adulthood remains difficult for a number of reasons, here we refer to older adults as those aged 50+, in line with existing literature (Rader and Wash, 2015), including a more inclusive age bound allows for a greater likelihood of diversity in our older adult sample.

As lockdowns eased, people were once again able to go out and about in society, but the rapid rise in contactless digital interactions brought challenges for many older people (Kotkowski and Polasik, 2021). Cash transactions were discouraged, paper menus had all but disappeared, and QR codes became ubiquitous; not only for COVID-related “checking in” processes in the hospitality industry, but also for digital (and therefore socially distant) communication with waiters, etc. Little is known about the experiences of older people during this period of emergence from lockdown, but it is possible that further social and digital inequities were propagated at this time, and that is the focus of the present study.

We explored the experiences of older adults in this increasingly contactless digital world, asking questions about the particular challenges they faced and about how they dealt with these challenges. In doing so, we learned something distinctive about the factors that make people experience exclusion as an acute phenomenon and noted the ways this could drive behavior change around technology use. Specifically, in our study, and in the literature below, we ask to what extent the rapid digital changes made following lockdown periods disadvantaged some older adults, how this disadvantage was experienced, and whether this experience influenced their desire to acquire new digital devices and skills.

## 2. Background literature

### 2.1. Older adults and the digital divide

There has been much discussion about the digital divide and the existence of a “digital underclass” (Helsper and Reisdorf, 2017), referring to groups of citizens who have a limited digital voice and limited access to online services. Older adults are one demographic that risk falling into this underclass, often exhibiting the “three levels” of digital deprivation: limited or no Internet access, low digital literacy, and relatively poor agency when engaged online, resulting in adverse offline consequences (van Deursen and Helsper, 2015; Schreurs et al., 2017; Hunsaker and Hargittai, 2018; van Deursen and van Dijk, 2019).

A number of initiatives have been designed to promote higher levels of digital inclusion (Reisdorf and Rhinesmith, 2020) by improving digital literacy (Radovanović et al., 2020) and offering greater social support (Asmar et al., 2020). Despite this, digital inequalities remain pernicious: particularly in the older adult community. Recently, researchers have begun to ask whether this is necessarily an *inequality* problem relating to digital access and/or skills, or a matter of *informed choice*, given that many older adults have expressed the view that they are content to live their lives offline, challenging the prevailing view that Internet access inevitably delivers benefits, and that poor access has adverse consequences (Scheerder et al., 2017). Put simply, researchers have recognized that some individuals simply do not wish to go online and see no advantage in doing so (e.g., Wyatt, 2003; Satchell and Dourish, 2009; Page et al., 2018), while others have a limited online presence, but see no need to improve their basic skills (Bardach et al., 2021). In other words, there are strong motivational reasons that can explain the limited use of digital technologies which go beyond access and literacy barriers. This distinction is important in our work, as the COVID-19 pandemic has arguably transformed the motivational grounds for digital non-use, given the sudden removal of much of the physical and social fabric of everyday living. If friends can no longer drop by, or businesses no longer accept cash payments, then surely the benefits of online interaction begin to outweigh the costs, even for those individuals who had previously eschewed online activity.

Mossberger et al. (2015) noted that technologies cannot be separated from the social systems and processes within which they are embedded, and that motivations to go online will inevitably depend upon the extent to which digital access can determine one’s ability to fully participate in society. Helsper (2017) takes this further, arguing that some of the motivational factors underlying the digital divide can be best explained by Relative Deprivation Theory, an established theory that argues a more nuanced and relative understanding of “deprivation” in terms of people’s subjective assessment of their own personal circumstances. The theory suggests that relative disadvantage exists when people perceive themselves to be (unjustifiably) disadvantaged or different in comparison to others in a certain situation. Helsper argues that this idea should be central when understanding digital inequalities because people will only feel that they need to become digitally active when this feeling of being unjustifiably disadvantaged becomes prominent. The decision to disengage from digital life can also be self-perpetuating, i.e., a number of recent studies have shown that a form of self-directed agism can emerge, whereby people come to believe that they are simply too old to learn new digital skills (Kottl and Mannheim, 2021).

### 2.2. Digital challenges for older adults during the COVID-19 pandemic

During the COVID-19 pandemic, older adults found themselves particularly disadvantaged: experiencing the ‘triple jeopardy’ of being (1) more likely to develop serious conditions and experience higher mortality; (2) less likely to obtain high-quality information or services online; and (3) more likely to experience social isolation and loneliness (Xie et al., 2020). They struggled to get good access to digital healthcare (Litchfield et al., 2021) and were less likely to book healthcare appointments online or to access online banking (Centre for Ageing Better, 2020). They were also less likely to use digital forms of communication (video calls, text messaging, social media, and online games) to compensate for the restrictions of social distancing and lockdown (Nguyen et al., 2021), with only 20% of those aged 65 and older participating in online social gatherings with friends or family (Vogels, 2020).

In purely demographic terms, the situation for older adults was bleak: a situation compounded by the fact that older adults were vilified for their vulnerability, and a new form of agism appeared as people began to feel that lockdown restrictions were only needed to protect the old, perhaps most clearly signaled by the widespread use of the Twitter hashtag #BoomerRemover (Fraser et al., 2020). Yet, despite these challenges, there was also a sense that older people displayed great resilience during this time, possibly able to draw upon a richer set of life experiences to make sense of the changing landscape (McKinlay et al., 2021) and able to resign themselves more easily to the social restrictions that frustrated younger people (Lebrasseur et al., 2021).

The extant qualitative literature provides a more nuanced view of the lived experience of older adults during that time, and offers a rich source of information about the ways that older adults responded to the pandemic. For example, Mikal et al. (2021) followed 22 older adults for 6 weeks during the pandemic, using longitudinal qualitative surveys as a means to study digital engagement and mental health outcomes. They found that older adults effectively used social media for entertainment and education, but were less comfortable accessing online resources, and struggled with larger social media communities, preferring one-to-one communication. Talbot and Briggs (2022) interviewed 19 older adults with mild-to-moderate dementia, noting that most participants could use digital means to combat the stresses of the pandemic. Many used video and social media to boost connectedness, while some engaged in digital volunteering and/or used the Internet to acquire new skills; however, these activities were sometimes mentally exhausting for this particular group. Perhaps most telling, Fuller et al. (2022) interviewed 76 older adults aged 70–97 and found a notable difference in technology use dependent upon both age and attitude. Those in their 70s and early 80s were more willing to use video-technology to keep up with friends and family, but across all ages, there were some people who consistently reported reluctance to use digital means. The authors noted that “*many indicated a decisive and firm commitment to not adapting new technologies at their age, even if they could imagine the benefits,*” with some finding it too challenging, and systems such as Zoom seen as simply too much of a hassle.

Lebrasseur et al. (2021), in their rapid review of the experiences of older adults dealing with COVID-19, noted that it was difficult to treat older people as a homogeneous group, as their individual circumstances varied enormously. Some of the most vulnerable found themselves isolated, yet reliant upon others to deliver basic necessities. Others, however, were able to shop, socialize, or gain medical attention online. Clearly, there were major differences in the contexts in which older adults were asked to cope, with huge variation in both personal social networks, economic status, digital literacy, and attitude to technology use (e.g., Tabassum, 2020; Fuller et al., 2022), with those falling on the “wrong side of the senior digital divide” being much more likely to experience adverse effects of the pandemic (Robinson et al., 2020). In short, older adults showed great diversity in their ability to respond to digital upheaval, with some reporting positively about the transition to online activities as a means of coping (Rotenberg et al., 2021), while others doubled-down on their beliefs that technology solutions were not for them (Fuller et al., 2022).

### 2.3. Emerging from lockdown

One of the key things that happened following lockdown was that digital transformation moved out of the home and into the public sphere, with businesses rapidly introducing new contactless digital measures that enabled customers to buy products or services, while maintaining some form of social distance (Iqbal and Campbell, 2021). These new measures excluded those without smartphones, good connectivity, or the necessary digital skills. In other words, they excluded many of those older adults who, intentionally or unintentionally, lacked the digital means to engage. An interesting aspect of this new development was how quickly previously in-person interactions suddenly became digital. For example, on entering a restaurant, the process of being shown to a table and given a paper menu was swiftly replaced by the requirement to “check-in” *via* a QR code, and then order (and pay) online. The default, certainly in the hospitality industry, became a digital exchange *via* smartphone (Kohli and Rohtak, 2021), meaning that those who were without a smart device, or who were unfamiliar with the relevant apps and services, struggled. Critically, this struggle took place in the public sphere, while others looked on, and so our aim was to understand how such moments were experienced, what kinds of access to goods and services were denied to our older adults, and whether there were any longer-term consequences, e.g., in terms of acquiring new devices and/or digital skills.

## 3. Materials and methods

Ethical approval for this study was obtained from the psychology ethics board within the University of Northumbria at Newcastle on 19/10/2021.

### 3.1. Study design

We employed a multi-method qualitative approach, combining an online qualitative survey with online one-to-one interviews. Online, qualitative surveys are increasingly recognized as a means to generate qualitative data at scale. Braun et al. (2021) have argued that such surveys act as a “wide angle lens” on a relatively under-researched topic, ensuring sufficiently diverse voices are heard. They also noted that answers to online surveys can be brief, which is why qualitative surveys benefit from the supplementation of interviews that allow identified issues to be probed in greater depth.

### 3.2. Participants

The online survey was developed on Qualtrics (Qualtrics, Provo, UT, 2018) and administered to a sample *via* (Prolific, 2014) an online survey company. A soft launch of approximately 10% (*n* = 10) of the overall sample was conducted to ensure that the survey contained no errors, as well as to establish an appropriate payment for participants. The average completion time of the overall sample was just under 14 min. Participants were remunerated with £1.88 for taking part, a figure deemed “good” by prolific. In total, 128 participants accessed the survey. Of these, 93 completed the survey in full, giving a completion rate of 72.7% and a sample size in keeping with those suggested by Braun et al. (2021). Following the survey, one-to-one interviews were held with a sample of older adults (*n* = 9, 10% of survey number), giving us data for 102 participants in total. Demographics for the 93 survey participants (Table 1) and 9 interview participants (Table 2) can be seen below.

**Table 1:** Demographics for online survey respondents (*n* = 93).

Demographic	Descriptor	Percentage
Sex (% Ratio)	Male: 33 (35.5%) Female: 60 (64.5%) Other 0 (0%)	
Age (SD)	Mean overall: 55.63 (4.84)	
Age range	Minimum: 50 Maximum:76	
Employment status	Full time	33 (35.5%)
	Self employed	19 (20.4%)
	Part time	17 (18.3%)
	Retired	16 (17.2%)
	Unemployed/Seeking	4 (4.3%)
	Other:	
	Unable to work	2 (2.2%)
	Homemaker	2 (2.2%)
Relationship status	Married	56 (60.2%)
	Single	14 (15.1%)
	Divorced	8 (8.6%)
	Living with partner	7 (7.5%)
	Separated	4 (4.3%)
	Windowed	3 (3.2%)
	Civil partnership	1 (1.1%)
Ethnicity	White: English/Welsh/Scottish/Northern Irish/British	42 (45.2%)
	White: Any other background	28 (30.1%)
	Any other ethnic group	8 (4.3%)
	Prefer not to say	4 (4.3%)
	Black/African/Caribbean/Black British: African	2 (2.2%)
	White: Irish	2 (2.2%)
	Black/African/Caribbean/Black British: British	2 (2.2%)
	Asian/Asian British: Indian	2 (2.2%)
	Asian/Asian British: Chinese	1 (1.1%)
	Any other Asian background	1 (1.1%)
	Mixed/Multiple ethnic groups: White and Black African	1 (1.1%)
Qualifications	PhD or equivalent	5 (5.4%)
	Master’s Degree or equivalent	18 (19.4%)
	Postgraduate Diploma or equivalent	9 (9.7%)
	Undergraduate Degree or equivalent	26 (28%)
	A-Level or equivalent	17 (18.3%)
	GCSE/O-Level or equivalent	12 (12.9%)
	No formal qualifications	6 (6.5%)

**Table 2:** Demographics of interview respondents.

**Demographic**	**Detail**
Age	Average: 65 (64, 67, 63, 65, 63, 61, 67, 75, 60)
Gender	5 Male, 4 Female
Living arrangement	1 lives alone, 5 live with partner only, 1 lives with friend, 1 lives with partner and two children
Ethnicity	9 White

As noted above, as part of a mixed qualitative methods approach, nine further individuals (amounting to 10% of participants) were interviewed online (*via* Zoom). There were four women and five men, aged between 60 and 75. Six were married, two were single, and one was in a relationship (living separately).

### 3.3. Materials and procedure

In the online survey, participants were asked to recount a recent face-to-face experience where they had interacted with new, post-COVID digital interactions. Participants were informed that the research was particularly interested in negative or frustrating experiences, as although digital interactions may be positive for some, such cases are not useful when attempting to understand the possible repercussions for digital exclusion of newly implemented interactions. Participants were asked to provide a range of information including: where the interaction took place, what devices were involved, who else was involved, what happened, and how they felt about the situation. This technique of asking participants to describe a lived experience, but then prompting for detail, is recommended by Braun et al. (2021) to ensure that participants give sufficiently rich responses.

For the one-to-one interviews, participants were again invited to discuss their recent experiences of digital technology when emerging from lockdown, but with additional probing in relation to the context of their interactions. They were also asked further questions about their use of technology throughout lockdown, as well as the extent to which they felt that new systems and measures would be “here to stay.” In the results section below, these participants are labeled with (I) signaling interview.

## 4. Analysis procedure

The experiences reported by our older adult survey sample were compiled and printed into paper format. Authors 1 and 2 (both very experienced in participatory digital work with older adults, one aged over 60) then conducted a thematic analysis in line with Braun and Clarke (2006) guidelines to identify recurring themes within the data through a process as follows. Authors 1 and 2 familiarized themselves with the data, looking in particular for vivid and compelling stories (as recommended by Braun et al., 2021). Author 1 generated the initial codes, which were then reviewed by authors 1 and 2 in a face-to-face paper-based sorting exercise. First, the authors reviewed the appropriateness of the codes in relation to the quotes, to ensure agreement that the codes were appropriately representative of the content of the experiences outlined. Codes were revised and agreed upon, where appropriate, through discourse. Following a review of the codes, the authors identified clusters of thematically similar codes, while iteratively revising groupings for the most appropriate fit. For the interview transcripts, the codes used for the survey were retained and supplemented with additional codes, initially suggested by author 3 (experienced in participatory work with older adults) but then reviewed by author 2.

## 5. Results

We asked for difficult experiences and overwhelmingly we were presented with detailed stories of exclusion and failure, caused by either intrinsic (personal) factors (e.g., do not have devices, do not want to use services, or do not know how to use them) or extrinsic (organizational) factors (e.g., poor quality Wi-Fi, poor usability) that impeded the success of newly implemented digital procedures. This division echoes that described by Wyatt (2003) when describing the reasons for “non-use” of technology as well as the factors identified by Morrison et al. (2021) which explain reasons for older adults’ disengagement from cybersecurity practices. Essentially, here, we use the division to help elucidate the different *sources of exclusion*, which are captured in Table 3 and elaborated in the text.

**Table 3:** Sources of exclusion in post-lockdown interactions.

Intrinsic (personal) sources	Extrinsic (Environmental) sources
Limited access to required technology (e.g., do not carry a smartphone, no Internet data)	Poor Wi-Fi access and/or mobile phone reception
Devices do not offer required functions (especially older devices)	Poor usability of forms or procedures
Reluctance to use the technology (e.g., the test and trace app)	No flexibility in procedures (e.g., no workaround if digital fails)
Limited knowledge of how to use technology (e.g., QR codes)	

### 5.1. Intrinsic (personal) sources of exclusion

It has been noted that many older adults exclude themselves from the digital world, arguing that they see no pressing need to access online resources, nor any advantage in doing so (Wyatt, 2003; Satchell and Dourish, 2009). In our data, we can see the various forms that this exclusion takes, but also note the way that personal decisions (such as not carrying a smartphone) can suddenly become problematic.

#### 5.1.1. Limited access to required technology (e.g., does not carry a smartphone, has no Internet data)

Many post-lockdown digital innovations were designed to allow some physical access to places, goods, and services while maintaining social distance and reducing person-to-person contact. For example, bars and restaurants typically implemented a QR code system whereby menus could be sourced online, and payment could be made electronically (such as through an app or website). However, such digital interactions were not available to all. Some participants reported difficulties because they did not own or carry a smartphone:

[24] - When I was in a restaurant, they said they could not give us menus and we were asked to scan the QR code with our phones to see the menu (we have never heard of that, so we were not prepared). My partner and I did not have our phones with us, because we wanted to disconnect. We could not see the menus, but we were not too frustrated. The situation was resolved because we both ended up ordering what the waiter recommended.

[79] – [my wife] had gone to the post office to carry out some operations. Upon entering, an employee of the office asked her to show the QR code of the booking and the green pass, that is a QR code that certifies the Covid vaccination. My wife had neither and so she had to leave the office.

[3i] I went to one restaurant where they wanted me to order on their QR code but when I didn't have a smartphone. They brought me their iPad to use to order. Given the whole point was Covid protection, they were happy for me to pore over their iPad but wouldn't hand out paper menus, that didn't make sense.

Although technology adoption and acceptance is steadily increasing in older adult populations (Mitzner et al., 2019), it remains lower than in other demographic groups (Paul and Spiru, 2021). As a result, many older adults still do not own their own smartphone, with a recent Pew report noting only 61% of smartphone ownership in those aged 65 and older (Faverio, 2022). For those older adults that do own smartphones, their usage is likely to be lower than younger populations (Li and Luximon, 2018; Mariano et al., 2021) and as such, they may not feel the need to always carry them. Inevitably, this meant that some of our participants had to seek help from others, asking waiters for verbal recommendations, or use alternative devices to view the menu or pay.

#### 5.1.2. Device does not offer function

For those with smartphones, there were sometimes issues in accessing the relevant function which meant that the interactions were far from seamless. For some, having older or faulty devices led to situations where the digital interaction was not possible, or put them in situations where friends with better connectivity were successful where they failed:

[9] - I went out to eat at a restaurant and they require you to pay via a cashless payment system called Zapper. I had to use the app on my phone which is linked to my bank card and scan the QR code printed at the bottom on the receipt. It was me and my family at the table. My camera is slightly faulty and occasionally can be blurry, therefore it was hard for me to scan the QR code. It was very embarrassing as the waiter stood closely waiting to be paid as my phone struggled to scan the code. I felt stressed and embarrassed as this is not a great situation.

[38] Most negative experiences were when visiting coffee shops that had track and trace apps. The NHS app only worked for IOS above version 13 (Older iPhones such as the iPhone 6S are not capable of operating at IOS version 13 so this caused an immediate problem with using the app). The Government believe (wrongly) that everyone can afford the latest smartphone and build their app accordingly. This makes the vast majority of people unable to use the app effectively.

For others, technology failures led to refusal of entry, or frictions when attempting to enter some venues:

[17] - The person that greeted us asked to sign covid paper and show vaccine certificate on our phones. I showed him my cert and when it was my wife's turn, she had a problem loading it in. The worker didn't let us in because we didn't have proof she was vaccinated. I felt very annoyed and frustrated because it wasn't her fault her phone didn’t work.

[18] – We had difficulties when checking into a hotel that required proof of a negative Covid test for myself and partner. We both had the NHS app, but one [of us] couldn’t access it. We had the alternative of showing it from the test provider, but the email itself wasn’t sufficient. We had to find the log in details for the company, and then download the result, and then forward it on by email to the hotel with a screenshot. This took over half an hour and was very frustrating.

Quite often, these protracted exchanges involved complex operations conducted on a smartphone. While it is not uncommon for younger users to use their mobile phone to make complex transactions (such as making travel arrangements), older adults are often more uncomfortable with such processes (Pangbourne et al., 2010; Jamal and Newbold, 2020).

#### 5.1.3. Reluctance to use the technology/app

The rollout of new digital measures was rapid, and some innovations were controversial. For example, in the United Kingdom, the government recommended the universal use of the “test and trace” app that would track location and monitor for proximity to person, or persons, who later tested positive for COVID-19. While use of the app was discretionary, restaurants and bars were required to keep a record of customers and most relied on the test and trace app as a means of doing this. Unfortunately, the test and trace app was not always reliable, leading to stories of a “pingdemic” whereby citizens were told they had been in proximity with someone with COVID-19, even when this was highly unlikely. For that and other reasons, some people did not use the app, and this could lead to problems:

[83] - On entering the restaurant … I didn't have the NHS app. I said I will leave my name and contact details which were accepted at other places, but the waiter insisted the app must be downloaded to scan the barcode. After a few minutes debating that it wasn't a legal requirement to have the app, she refused me entry to the restaurant. I felt very frustrated and peered pressured into doing something that wasn't required by guidelines and felt embarrassed being treated like this in front of customers.

[106] I have been really frustrated at having to scan track and trace into restaurants, in fact I refused to have it on my phone after a bad experience. We were greatly delayed entering the restaurant as I needed to register first, the restaurant was completely empty and we sat outside at the end there was then no way to check out. Later that day someone came up positive, so we had to self-isolate even though we were there hours before!

[9i] I never downloaded the track and trace … I wasn't giving my money or details to anyone in the government. But I was surrounded by people who downloaded it who were pinging all the time.

More typically, those unable or unwilling to download apps such as NHS Test and Trace faced minor inconveniences such as having to “sign in” manually to venues, something which may now be considered favorable in light of some citizens’ concerns around the privacy and security implications of such applications and others (Akinbi et al., 2021; Sowmiya et al., 2021).

#### 5.1.4. Do not know how to use the technology (digital literacy)

One participant, quoted previously, said that they had “never heard” of scanning a QR code to see a menu. This was common, with many individuals unsure of how to use the code:

[31] - It happened at Ben and Jerry's in Vermont. There were about 20 people in line on a hot day to get ice cream. There was a QR code posted on the wall. No explanation. Just a code. Most young people know what this means and how to use it. Why would that be assumed that everyone knows how to do this? So, as I tried to figure it out, my 17-year-old was visibly and vocally embarrassed that I hit a wrong button. This, in turn, embarrassed me. It would not have taken much time to have an explanation on the code.

[86] - When I entered the shopping mall, a worker of the mall asked me to use QR code by taking a picture of it with my smartphone camera. I had no idea what a QR code is or how to use it. It was not explained what it is for and I felt very stupid. By some reason it did not work (I still do not know what it was supposed to do).

[6i] - We had never done it before and didn't know how to do it, we got stuck and had to call the guy over who was really busy rushing around trying to take things to people's tables. He was helpful but you thought he doesn't have time to be faffing on with old people's phones, you felt like you were a couple of dinosaurs sitting there with this young person having to show you how to use your phone.

Such observations resonate with the classic age-based view of the “digital divide” as being primarily around digital literacy (Friemel, 2016; Helsper and Reisdorf, 2017), leading to a range of digital inequalities (Hill et al., 2015; Robinson et al., 2015, 2020). What is interesting here is how a seemingly simple, but ubiquitous change (information exchange *via* a QR code) could be so divisive. In some cases, our participants acquired this new skill rapidly, but not without some initial discomfort.

### 5.2. Extrinsic (organizational) sources of exclusion

In many cases, our participants were unable to act because of external problems, with some of the most common issues being an inability to gain access to the Internet (because of poor Wi-Fi or phone reception), which was particularly frustrating when a restaurant or bar asked them to download a dedicated app. At times, they were able to complete these actions, but then found usability problems when interacting with the relevant site or service.

#### 5.2.1. Poor Wi-fi access and/or mobile phone reception

Connectivity issues were frequently reported by participants. Attempts to access vaccination certificates and company-specific apps, such as those used to order food and drinks, often failed due to a lack of mobile signal, or being outside of Wi-Fi range. At times, this led to institutions using mitigation strategies such as paper slips to track customers. Others refused citizens entry leading to them being excluded due to their lack of access.

[67] - The pub had the NHS track and trace app outside, I do have a smart phone and I am confident with it. I scanned this several times, but nothing happened. I then realized that there wasn't a signal.

[89] Arriving to the restaurant, the staff asked for both [Covid Vaccination] certificates, my Mom showed hers on paper and it was all good, but when he tried to scan my certificate with the app he had on his mobile phone, it failed, it couldn't read it and it gave an error. Therefore, we could not go inside the restaurant!

At times, the lack of connectivity led to difficult or awkward situations, especially when some members of a party were unable to access relevant apps, but others had a better phone signal, or when some struggled to use cashless means of payment.

[19] – I met with friends at an outdoor restaurant. Needed to order using an app. Although I am confident using technology, I struggled to do it. Signal was bad, couldn't order so waiter took our order. Made me feel silly as others in our party were able to order quite easily.

[54] - At Nando’s the menu is via a QR code. On trying to access the QR code, I was unable to, as there was no internet access. This proved difficult to order. I was with a friend who also had the same problem.

#### 5.2.2. Requirement to download additional apps

Participants in this study discussed several ways in which the organizations had implemented new online procedures that were particularly burdensome or time consuming. Typical of these was the requirement to download a dedicated app to access a menu, order, or pay. Again, this was a source of annoyance or awkwardness, which was exacerbated when connectivity compounded these issues.

[34] - This situation happened in a restaurant; I was dining with my family when it was time to pay the check. I was the one paying, so I asked one of the waitresses to give me the check, I was surprised when she told me to take out my phone and open the restaurant's app. We were never told to download an app when we arrived, so I felt lost and annoyed as a result. It took me about 20 mins to pay the check and it just made me mad because if they would've told us to download the app it would've been easier and faster.

[37] - I was unable to order a meal in a restaurant without the help of downloading an app to scan and read their menu. It took a while to do this and as we were older than the crowd, the waiter was helpful, but they were busy, and I could see he was in a hurry. I did feel a bit behind the times.

[87] - Both me and my friend were sitting at table waiting for downloads trying to place order and work through a complex app which didn’t have special dietary requirements incorporated. Felt like we wasted half an hour giving details and ordering without even speaking to each other and all within arm’s length of the waitresses and a till!

#### 5.2.3. Usability problems (text too small/interaction poorly designed)

Failure of technology was not always the reason for difficulties using newly implemented digital interactions, however. For some users, the visual presentation of the application led to difficulties for users, this was particularly the case for those who struggle to see small content on a phone screen, something well established in older adult technology usability research (Zhou et al., 2014).

[66] - Well, the situation occurred when a friend and I went to eat at a restaurant, and we asked about the menu, and instead of telling us the waiter what they had, he told us that we had to use the mobile and see it via QR ..., seeing the menu from the mobile, as well as the prices was desperate … so we chose the first thing we found from the menu and then we left, the truth is that you feel somewhat helpless...

[59] - After waiting for a menu for a few minutes, a server came over and asked if I had decided what I wanted. I said I haven't seen a menu yet. She said we don't use them anymore … and said you have to scan the QR code with your smartphone. I was flabbergasted. With a bit of help to navigate the application on my phone, I was able to pull up the menu, but it was difficult to navigate while viewing something so small, that had to be magnified per section. I finally just ordered a standard item that I knew they would have.

In bars, restaurants, and other hospitality settings, these consequences may seem minor, such as ordering something recommended by a waiter, but as we hear below, the frustration, embarrassment, and shame experienced by many of our participants were significant.

#### 5.2.4. Reaction to change

Earlier, we made the point that older adults are a heterogenous group, showing a range of digital skills. Many of our participants found the new procedures manageable and this reinforced their own self-image as people who are digitally competent, or as people who could adapt to new procedures where necessary:

[56] - I am a person who is generally up to date with technology, the only thing that I had not used before was scanning QR codes, I thought it was somewhat complicated, but it ended up being easier than I thought.

[1i] I don't think before COVID I had ever paid for anything with my smart phone and now I do it without thinking.

Some of our participants had long since resigned themselves to the fact that digital was “not for them.” In some cases, they were simply resigned to restrict themselves to use only the most basic digital functions, living life without a smartphone or without apps. In many other cases, people had established procedures where a spouse or child was called upon to help cope with everyday digital demands and this simply continued post-lockdown.

[4i] I can barely function. I think I do the stuff that I have to absolutely be able to do …I feel like I hang on by my fingernails.

[5i] I find it’s a cashless society now. I have paid with a card but Julie, my wife, if we go out, she does all of that. She swipes things if we go for a meal’.

[3i] I get someone else to help me…Like most people, my wife is more adept at these things so now and again I will get her to solve the problem.

However, we did find many occasions when people were experiencing significant digital obstacles for the first time. Often, this exposure took place in a public environment, and led to our participants feeling helpless, stupid, angry, and embarrassed, sometimes resulting in a greatly reduced self-belief:

[9i] for the first time it has felt very ageing. For the first time I felt shit, I don't know how I got to be 60, but maybe there will come a time where unless I am on top of my smart phone or apps then I will just have to stay at home and be a hermit.

Sometimes, these uncomfortable encounters became motivators for change, with people recognizing the need to acquire further digital skills or invest in a new device, something in keeping with the relational deprivation arguments outlined above; however, participants were often resentful about the need for change.

[3i] Well, on one occasion I simply had to leave the bar… I just went to another bar that didn't do QR codes. Another time, after much persuasion, we realized we could get a drink from one of the bars but still couldn't get any food. We went somewhere else to eat. Ultimately, I went and got a smart phone.

[70] - I started to feel really stressed and embarrassed about the situation. A few minutes later, someone I knew came into the store and showed me what I had to do, and thankfully it all worked OK. I felt really stupid not being able to work this out, but now I know how it all works, so it was a way of learning.

[48] As a consumer I always used to buy things domestically but now I have learned to utilize online shopping. We are even forced to use phone to communicate with our doctors because of the situations. Establishments only take a limited number of people inside, for example to order food, I have been forced to get used to ordering online

[95] I was forced to download the app on my phone and not knowing how to navigate the app I became very frustrated and impatient. I eventually decided to walk home and later went through the app in my own time.

There was a strong sense from these accounts that some people were being dragged screaming into a digital world. Often, the accounts were accompanied by tales of suspicion about the relative security or privacy of the apps or services involved (Elueze and Quan-Haase, 2018) and at other times, people were simply resentful of the fact that they had been forced to join the “always on, always available” generation.

[27] - They asked me to pay by using my bank app and I didn't like it. Why? Because I'm not that good with technology so I was scared of getting robbed.

[8i] People want to know too much; I don't know what it is about the modern world but this didn't used to happen. It’s all about your data so if they can find out things about you, it’s my information it should be up to me to decide who uses it.

[3i] I never wanted to carry a phone where I could get email or Facebook and all the kinds of social areas that I work in, I didn't want to be using those. So, I just avoided putting myself in that position. Now I have a smart phone that does creep in, and I still find it annoying.

## 6. Discussion

Recent literature on older adults’ digital literacy is characterized by the recognition that they are a highly heterogenous group and that decisions to go online are highly context dependent, i.e., not solely determined by skill level. In a study of New York older adults, Quan-Haase et al. (2018) found a nonlinear association between skill levels and online engagement. They found that many older adults were simply prepared to “give it a go” without the requisite skills, while others became consumed with worries that digital media might overwhelm them, or simply waste their time. We found something similar in our sample, where some participants quickly embraced the changes enforced post-lockdown, whether or not they were familiar with the apps etc., while others struggled. As noted earlier, one challenging issue was the fact that these struggles often took place in a public domain. This public humiliation was seen, by some, as a reason to withdraw from technology use, while for others, it would be accepted as a challenge to be overcome. In this discussion, we unpack some of these different responses, taking the rapid need to learn new digital skills post-lockdown as our starting point, and trying to understand more about why this created a motivation to learn in some, but a desire to withdraw in others.

### 6.1. Too old to learn

There is a pervasive social construction of older adults as inept users of technology and many people simply feel that technology has passed them by Schreurs et al. (2017) wrote: *“Given the presence of a sometimes negative or mocking portrayal of older adults in the media, it is important for older adults to have support in obtaining digital literacy, as it would be easy to fall victim to the rhetoric that they are “inept.” (Pg 373).*

Feeling “inept” or deciding that one is too old to try something is a form of “self-directed” agism (McDonough, 2020; Köttl et al., 2021) that can directly impede learning and ultimately lead to a less fulfilling life. This self-directed agism (accompanied by feelings of shame about getting older) is known to influence older adults wellbeing and quality of life and is also associated with greater cognitive decline (Kotter-Grühn et al., 2015; Bodner et al., 2021). In a number of the accounts from our participants, we heard people refer to themselves as “dinosaurs,” or say they were simply “too old” to learn. Being publicly exposed as “digitally inept” however, was a particularly stigmatizing experience and was often accompanied by a sense of shame from those who internalized this label. Public failure in a digital sphere not only reinforces this social stereotype, but also taints the self-image in a way that, for some, led to the decision to stop trying. These socio-emotional aspects of digital engagement (see also Eshet-Alkalai, 2004; Haight et al., 2014) are critically important when we want to understand more about the reasons digital literacy remains a problem for many older adults. It is particularly critical when we recognize that those who wish to learn have to “expose” their poor skills to their peer network, in order to seek out friends and family who are able to help.

### 6.2. Willing to give it a try

It is useful to turn to those in our sample who faced, but overcame, digital exclusion to see if there may be lessons to learn here. In particular, our data may shine some light onto the so-called “digital paradox” described by Okun and Ayalon (2022) as follows: In order to learn, older adults need greater exposure to new technologies, but they are often unable to gain that exposure without the help of others. In the post-lockdown situation, we have described here that exposure was somewhat thrust upon them, and some simply did their best to cope with that. Nonetheless, we can see how important family and friends were at this point. Having access to a “warm network” of experts (see Hänninen et al., 2021) was often critical. In the data we describe, this network of individuals would sometimes be relied upon to take over, but in some of the more helpful scenarios, the warm experts were able to teach the new skills quickly and effectively. It was helpful, in these circumstances, that the people involved shared the same sense of frustration over poorly designed apps or poor-quality Wi-Fi, as this, in turn, moved the focus away from that sense of being digitally inept, into one of learning to cope with a swiftly changing world.

Though we draw a line between intrinsic and intrinsic factors in our reporting, it is important to note that in reality such factors are often intertwined, and promote, or are driven by, ongoing systemic inequalities. Recent research by Yang and Du (2021) for example, highlights how financial disparities between males and females lead to increased digital exclusion for female older adults. Having less spending power has clear connotations for digital equality (Soloman, 2002) through the ability to purchase, protect, or update technology, and with a continuing global gender pay gap (Bennedsen et al., 2019), such digital inequalities are likely to continue well into the future.

As well as generating inequalities within groups, social structures are also likely to heavily promote inequalities across demographic groups too. For example, many older adults, especially those who have worked on low incomes throughout their careers may reach retirement age without significant savings or pensions. For these individuals, the inability to buy technology may be seen as reluctance or unwillingness to conform to a digital revolution, despite the individuals’ actual motivations. Such circumstances are likely to lead to promote the stigma and ageist attitudes we refer to throughout this paper.

### 6.3. A call to action

In the introduction, we suggested Relational Deprivation Theory (RDT) as a means of understanding some of the motivational issues that underly the digital divide. A key critical construct here is *value legitimacy*: is it acceptable that there are different outcomes for different individuals and that the resulting unequal distribution of resources is legitimate (Davis, 1959; Janmaat, 2013)? This question becomes particularly interesting when the landscape suddenly changes and when new inequalities emerge. We know that many older adults do not engage with technology because they cannot see the benefit, i.e., they have low value expectations from technology use. But in the face of a sudden move to contactless exchanges *via* apps and QR codes, the value proposition in owning a smartphone, and having the skills to use it, changes.

Some people being turned away from a restaurant or finding that they cannot access a menu, or pay for their food is not a “legitimate” social disadvantage. It is not an acceptable new “‘societal norm” that older adults should be turned away simply because of the devices they own or their levels of digital literacy. RDT scholars would not expect such unfairly disadvantaged individuals to simply upskill themselves but would ask what steps society could take to address the problem. Helsper (2017) asks whether there are *mesocommunity processes* that could be put in place, leading to structural and sustainable changes in digital inequalities, stating: *“we do not yet know in which ways outrage at how the unequal distribution of digital resources disadvantages a particular community could lead to collective calls for action.” (p 234).*

It is not yet clear what societal or mesocommunity processes have been put in place as a result of the inequalities associated with the COVID-19 pandemic. There has indeed been “outrage” at some of the health inequalities that have come to light as the pandemic effectively exposed ‘fault lines’ within existing systems (Kawachi, 2020). The digital inequalities we have described in this paper are insignificant by comparison, but they are interesting nonetheless, not least because of the speed with which the landscape changed and digital fault lines became exposed. At the time of writing, this landscape has changed once again. QR codes and restaurant apps have faded away a little, and there has been a return to at least a hybrid system where one can once again order or speak directly to a waiter or bartender. In future work, it would be interesting to note the extent to which changes in skill levels acquired during and immediately post-pandemic would be sustained, or indeed whether attitudes toward digital skill acquisition changed for good in some segments of the older adult population. For example, was there any significant change in relation to “self-ageism” and the belief that one is too old to learn? Did some people become more aware of the technological skills of their immediate peers and start to consider the ways in which their own mindset might put them at a disadvantage? Or were other contextual factors at play that meant that, for some, they could once again eschew technology as being simply unnecessary given their own lifestyle choices. Such questions could guide a more nuanced understanding of the actions society might take in relation to the somewhat pernicious digital divide.

## 7. Limitations and considerations

A possible limitation of this study is the potential for self-selection in our sample, i.e., that our participants are those who have enough digital literacy to engage with online surveying companies such as Prolific, and as such are likely to be more digitally proficient than their peers. Although we could consider this a weakness, it is highly likely that negative connotations of becoming digitally excluded by the rollout of new digital interactions are likely to be exacerbated even further in those with limited access to technology, or the requisite digital skillsets to navigate such interactions. As such, the implications for older adults outlined here are likely to represent only the “tip of the iceberg.” Future research is required to understand the extent to which such exclusion impacts the lives of those in such positions.

Our sampling was intentionally broad within this study, designed to access a wide range of experiences from our participants. We placed no boundary on participant nationality or locality but found interesting similarities in the experiences we gathered in spite of this. Given the qualitative nature of this study, such similarities are outside of the scope of this paper, but the research community would likely benefit from understanding how technological solutions to the COVID-19 pandemic varied across nations, especially when considering the possible implications of wealth and health inequalities.

To further access a wide range of experiences, we also sampled broadly in terms of age, using a 50+ age criteria. As mentioned earlier in this paper, a large array of criteria are used across the extant literature base when working with older adults. It is however important to highlight that technology use and acceptability is likely to range within the older adult population. The older adult population is arguably the most diverse group of users, ranging from early adopters (and early developers) to those who have, and always will be, reluctant users of technology. Acknowledging this variability through inclusive design (Clarkson and Coleman, 2015) is essential to ongoing efforts to include older adult users in the technology landscape, especially those who are keen to do so but who are underserved by policymakers and developers who assume a base level of knowledge and access which may not be as prevalent across all user groups.

It is also important to acknowledge that many of the issues highlighted in this paper are not only experienced by older adults. Digital inequalities span across a number of (particularly marginalized) groups and are exacerbated by intersectionality (Zheng and Walsham, 2021). As such, many of the issues we report here are not only experienced by older adults, but are driven by the systemic inequalities we refer to above. Identifying and increasing the transparency of the issues underpinning digital inequality is therefore one possible avenue to help counteract the self-directed ageist stereotypes experienced by older adults.

## 8. Conclusion

In this paper, we have described new forms of digital exclusion, particularly in the hospitality industry, that adversely affected older adults during the post-lockdown period. We have described how both extrinsic (access to devices and services) and intrinsic (possession of relevant skills and knowledge) factors could lead to older adult exclusion and generate feelings of anger, embarrassment, and shame. We interpreted our findings in terms of relational deprivation theory (wherein inequalities that were once acceptable are now deemed unjust) and also in terms of the limiting effects of self-agism. We also found evidence of digital mobility: Some people, in the face of sudden and seemingly unjust digital change, swiftly acquired relevant skills, provided they had ready access to “warm experts,” and could acquire the necessary self-belief.

## Data availability statement

The datasets presented in this article are not readily available because due to the identifiable qualitative nature of the data, raw data will not be made available. Requests to access the datasets should be directed to benjamin.a.morrison@northumbria.ac.uk.

## Ethics statement

The studies involving human participants were reviewed and approved by Northumbria University School of Psychology Ethics Committee. The patients/participants provided their informed consent to participate in this study.

## Author contributions

All authors listed have made a substantial, direct, and intellectual contribution to the work and approved it for publication.

## Funding

This project contributes towards the EPSRC funded Centre of Digital Citizens [Grant number: EP/T022582/1].

## Conflict of interest

The authors declare that the research was conducted in the absence of any commercial or financial relationships that could be construed as a potential conflict of interest.

## Publisher’s note

All claims expressed in this article are solely those of the authors and do not necessarily represent those of their affiliated organizations, or those of the publisher, the editors and the reviewers. Any product that may be evaluated in this article, or claim that may be made by its manufacturer, is not guaranteed or endorsed by the publisher.

## Supplementary material

SUPPLEMENTARY FIGURE S1 Question text provided to participants as part of the online qualitative survey.
