# Supplementary figures and images for: Life after lockdown: The experiences of older adults in a contactless digital world
Source: Front Psychol. 2023 Jan 13;13:1100521. doi: 10.3389/fpsyg.2022.1100521 (PMC9880547; doi:10.3389/fpsyg.2022.1100521)

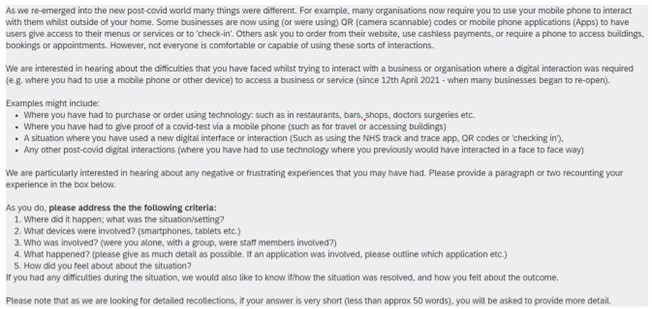

Supplement: SUPPLEMENTARY FIGURE S1 — Question text provided to participants as part of the online qualitative survey. [file Image_1.JPEG]
